# Determining body height and weight from thoracic and abdominal CT localizers in pediatric and young adult patients using deep learning

**DOI:** 10.1038/s41598-023-46080-5

**Published:** 2023-11-03

**Authors:** Aydin Demircioğlu, Anton S. Quinsten, Lale Umutlu, Michael Forsting, Kai Nassenstein, Denise Bos

**Affiliations:** Institute of Diagnostic and Interventional Radiology and Neuroradiology, University Hospital Essen, Hufelandstraße 55, 45147 Essen, Germany

**Keywords:** Diagnostic markers, Paediatric research

## Abstract

In this retrospective study, we aimed to predict the body height and weight of pediatric patients using CT localizers, which are overview scans performed before the acquisition of the CT. We trained three commonly used networks (EfficientNetV2-S, ResNet-18, and ResNet-34) on a cohort of 1009 and 1111 CT localizers of pediatric patients with recorded body height and weight (between January 2013 and December 2019) and validated them in an additional cohort of 116 and 127 localizers (acquired in 2020). The best-performing model was then tested in an independent cohort of 203 and 225 CT localizers (acquired between January 2021 and March 2023). In addition, a cohort of 1401 and 1590 localizers from younger adults (acquired between January 2013 and December 2013) was added to the training set to determine if it could improve the overall accuracy. The EfficientNetV2-S using the additional adult cohort performed best with a mean absolute error of 5.58 ± 4.26 cm for height and 4.25 ± 4.28 kg for weight. The relative error was 4.12 ± 4.05% for height and 11.28 ± 12.05% for weight. Our study demonstrated that automated estimation of height and weight in pediatric patients from CT localizers can be performed.

## Introduction

Somatometric parameters such as body height and weight are essential to routine clinical practice. They are central to the dosing of drugs and anesthetics and are often biomarkers needed for predictive scores or risk assessments^1–3^. They are also part of the radiological routine since body weight is critical for managing the radiation dose and influences the dosage of the contrast medium. Indeed, the European Commission recommends in its Radiation Protection No 185 that diagnostic reference levels (DRLs) be defined by weight groups for all body examinations^4^. Yet, most DRLs are still set based on age groups since, especially in emergencies, the body weight is not always available and must then be estimated by the medical staff. As the size and weight of children vary greatly with age, this can be challenging.

Usually, body height and weight are assessed multiple times and should be found in the medical information system, but at times it is difficult to retrieve them as they might be part of external and non-standard doctor’s letters, and recovering them would need high manual effort. Even if they can be found, they might have been recorded at differing times, e.g., months before the radiological imaging, rendering them less useful. Accordingly, methods for estimating body height and weight from radiological imaging have been studied as an alternative to direct measurement. For example, body weight and composition can be determined from a single CT slice^5^, from abdominal CTs^6^ or whole-body MRI scans^7, 8^.

Although not a modality on its own, CT localizers, also called CT scout views or topograms, which are overview images acquired with low radiation exposure, are available frequently since they are used to plan and delimit subsequent CT examinations, also in emergencies^9^. Despite their auxiliary role, they have shown to have a value of their own. Most prominently, it has been demonstrated that they can contribute to the diagnosis of the subsequent CT scan^10–12^.

In a recent study, CT localizers were utilized to estimate body weight in adult patients^13^. However, the potential applicability of these methods for pediatric patients remained unexplored. Hence, this study aimed to employ deep learning methods to automatically determine the body height and weight of pediatric patients using CT localizers.

## Results

**Figure 1:**
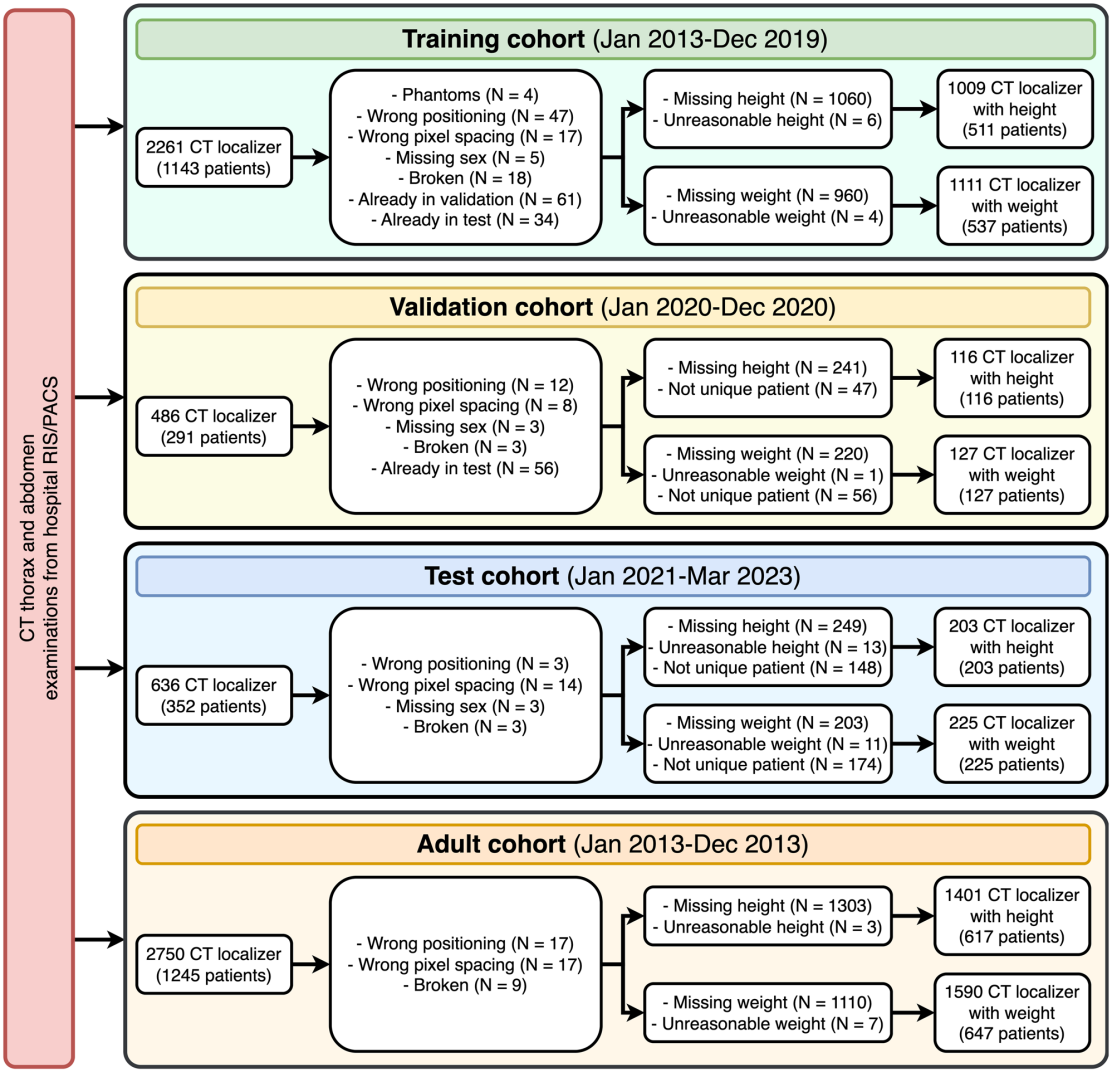
Patient flowchart.

**Figure 2:**
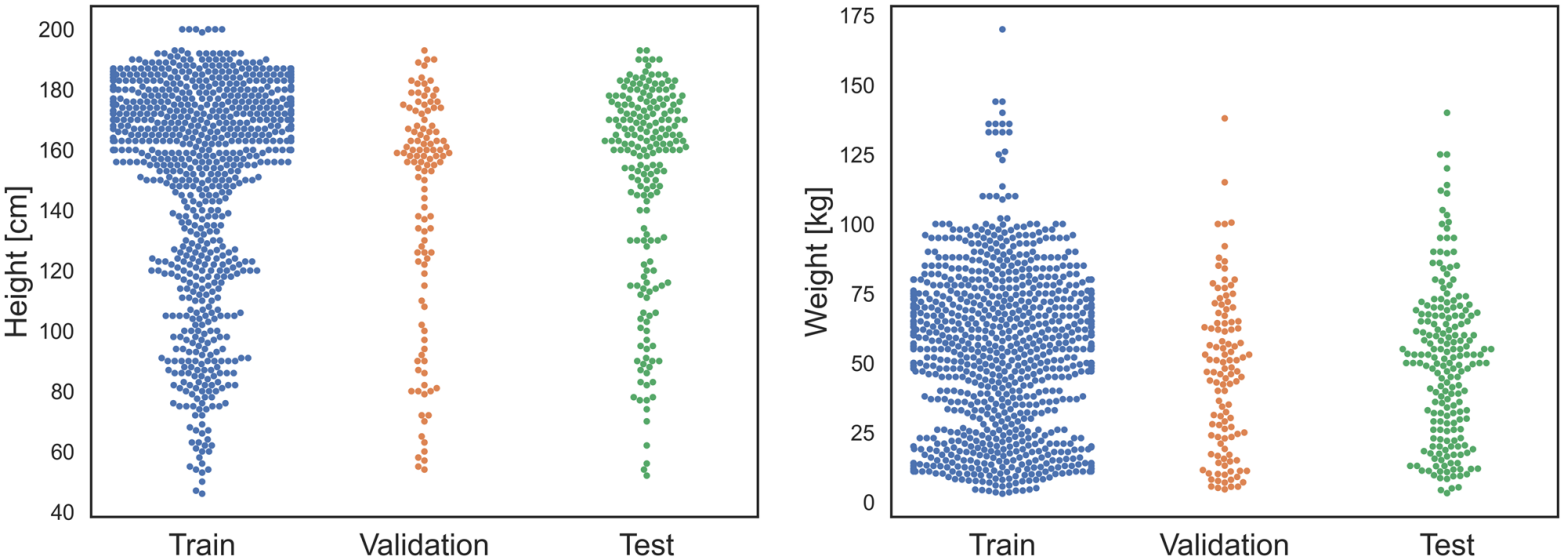
Distribution of body height and weights in the three patient cohorts.

A total of 1328 CT localizers from 830 patients were included for body height prediction, and 1463 CT localizers from 889 patients were considered for body weight prediction (Fig. 1). The mean age of all included patients was 13.3 ± 6.7 years (range: 1 month–21.0 years), with 375 females and 514 males (Table 1). The average body height was 1.47 ± 0.36 m (range: 0.46–2.0 m), while average body weight was 48.0 ± 27.2 kg (range: 3.0–144.0 kg). For model development, the CT localizers were split into a training, validation, and test cohort. The distribution of heights and weights between these three cohorts was relatively similar (Fig. 2). The demographics of the adult training cohort can be found in Table S1, Supplementary Information.

**Table 1:** Demographics of the patient collective.

	All (N = 889)	Training (N = 537)	Validation (N = 127)	Test (N = 225)
Gender (Female)	42.2% (375/889)	42.3% (227/537)	51.2% (65/127)	36.9% (83/225)
Height (m)	1.47 ± 0.36	1.47 ± 0.36	1.41 ± 0.38	1.50 ± 0.34
Weight (kg)	48.0 ± 27.2	48.2 ± 27.0	45.8 ± 27.5	48.7 ± 27.6
Age (years)	13.3 ± 6.7	13.3 ± 6.8	12.8 ± 7.0	13.5 ± 6.5

“All” summarizes all three cohorts.

### Validation results

Three network architectures, the EfficientNetV2-S, the ResNet-18 and the ResNet-34 were optimized once for the pediatric data and once for the training data together with the adult patients. Using only the pediatric data, the best network obtained an MAE of 4.77 ± 4.64 cm for body height and 3.93 ± 4.23 kg for body weight on the validation cohort, with a correlation coefficient of 0.985 and 0.978, respectively. The network showed relative errors of 3.69 ± 3.97% for height and 11.96 ± 14.3% for weight. Adding the adult training data improved the performance slightly: on the validation set, the MAE for body height was 4.36 ± 3.50 cm and 3.71 ± 4.11 kg for body weight. The correlation coefficients were R = 0.990 and R = 0.980. Accordingly, MAPE was 3.52 ± 3.56% and 9.85 ± 11.16%, respectively. Therefore, the network using adult patient data during training was selected as the best-performing model.

**Figure 3:**
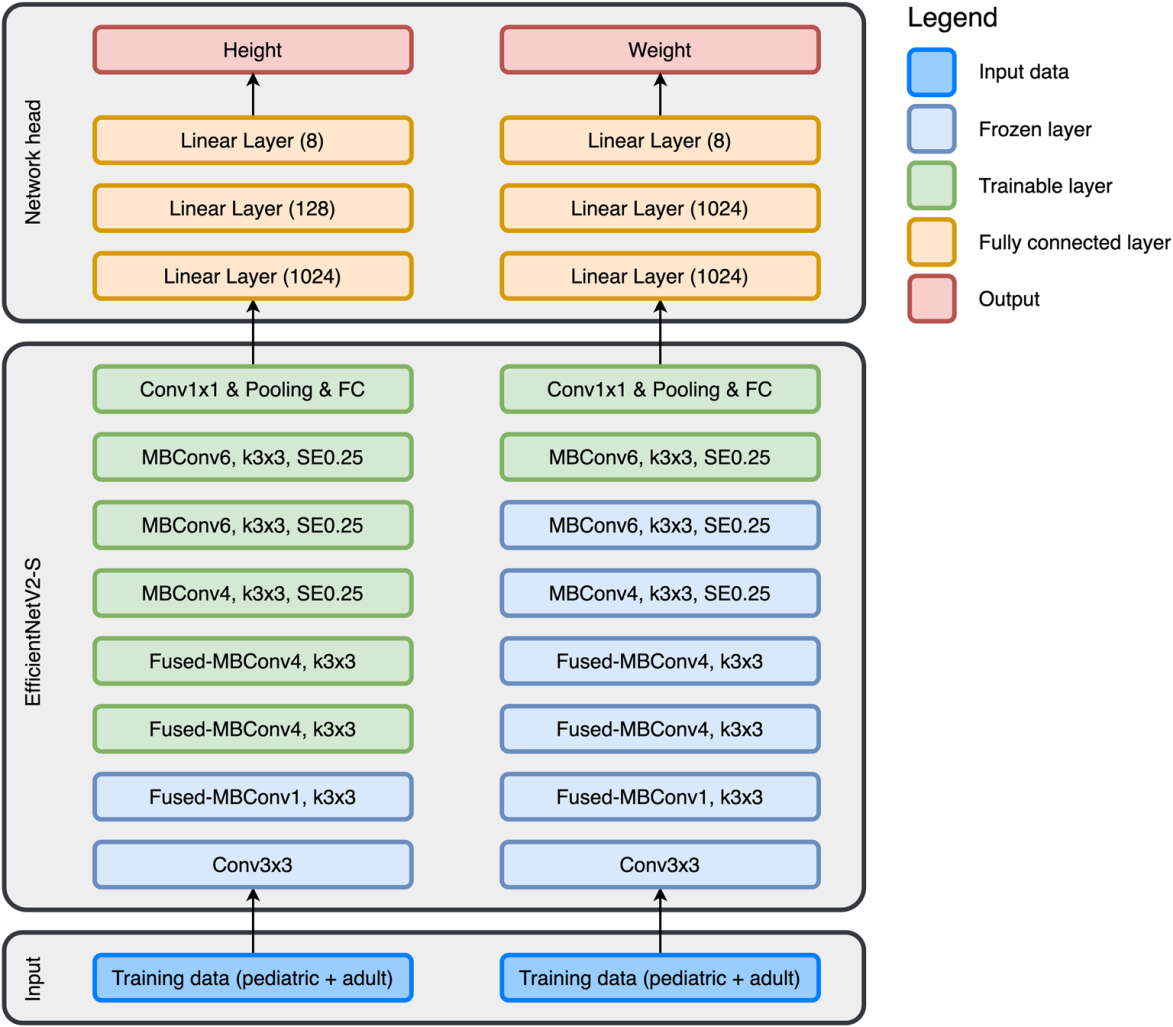
The architecture of the best-performing networks.

Model development used a hyperparameter optimization framework that tuned the pretrained network architecture, the layer sizes of the network head, the number of layers with weights frozen, and the learning rate. In both cases, for body height and weight, the pretrained EfficientNetV2-S was the best-performing network. For predicting the body height, the network performed best when the network head layers sizes were chosen to be [1024, 128, 8] (Fig. 3), the first five stages of the network were frozen, and the learning rate was set to 2.5 × 10^–4^, with a scheduling that multiplied it with 0.8 every 21 epochs. Learning proceeded until epoch 51, where no more progression could be seen.

Similarly, for the best prediction of body weight, the head layer sizes were set to [1024, 1024, 8], the first two stages (0 and 1) were frozen, and the learning rate was set to 1.0 × 10^–4^, multiplied by 0.5 every 19 epochs. The training was stopped at epoch 31.

### Test results

In order to exploit the data most efficiently, the best-performing models were retrained on both cohorts, i.e., the training and validation cohorts. Training proceeded with the optimized parameters and was conducted as many epochs as during optimization. These two models were deemed final and were then evaluated once on the independent test data.

**Figure 4:**
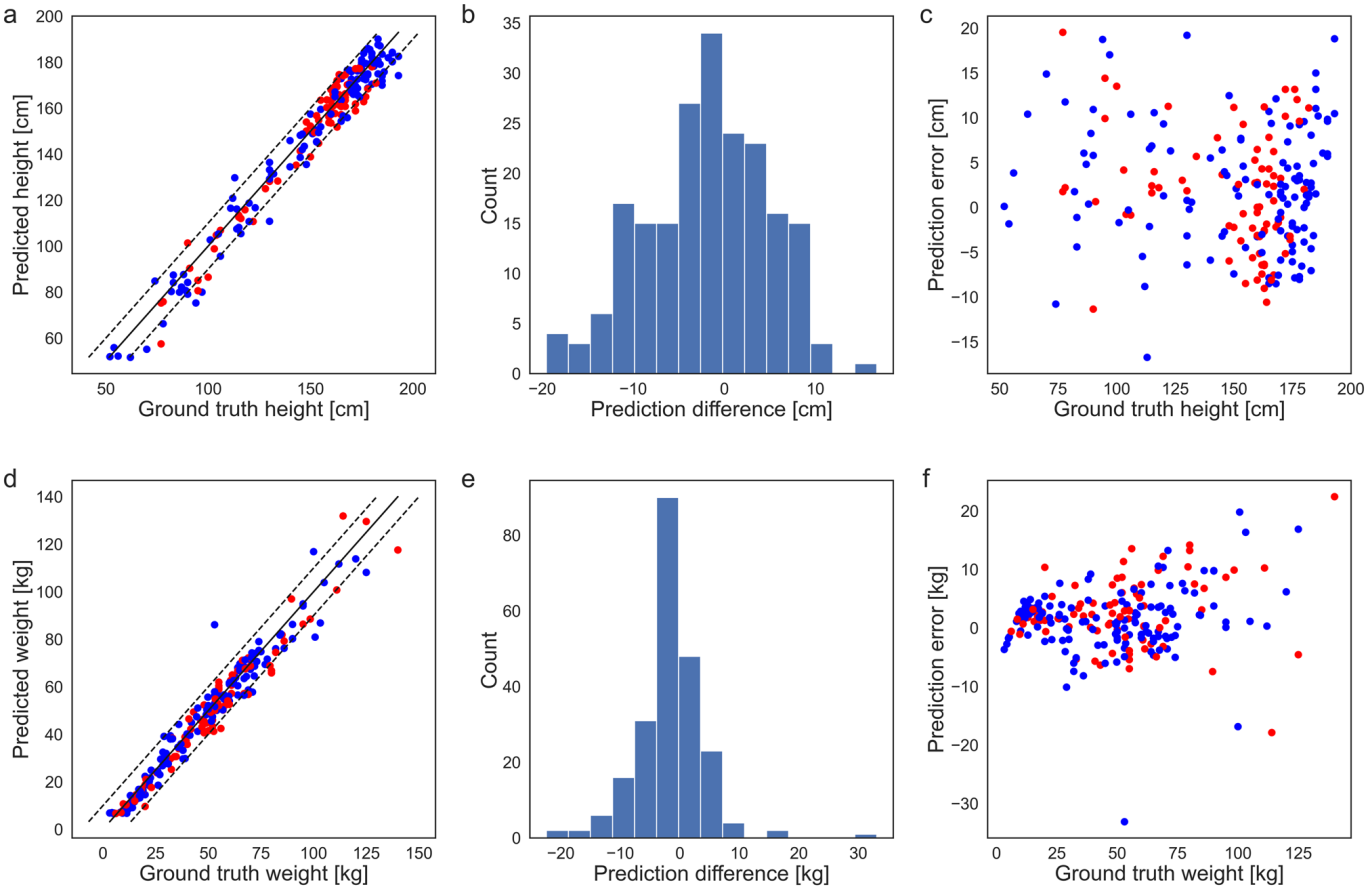
Visualization of the predictions of the final models on the independent test set (**a**) Scatter plot of height predictions versus ground truth. Red dots correspond to female patients, blue to male patients. The dashed lines mark the limits of ± 10 cm. (**b**) Histogram of the height prediction errors. (**c**) Scatter plot of height predictions errors versus the ground truth. (**d**) Scatter plot of weight predictions versus ground truth. Red dots correspond to female patients, blue to male patients. The dashed lines mark the limits of ± 10 kg. (**e**) Histogram of the weight prediction errors. (**f**) Scatter plot of weight predictions errors versus the ground truth.

**Figure 5:**
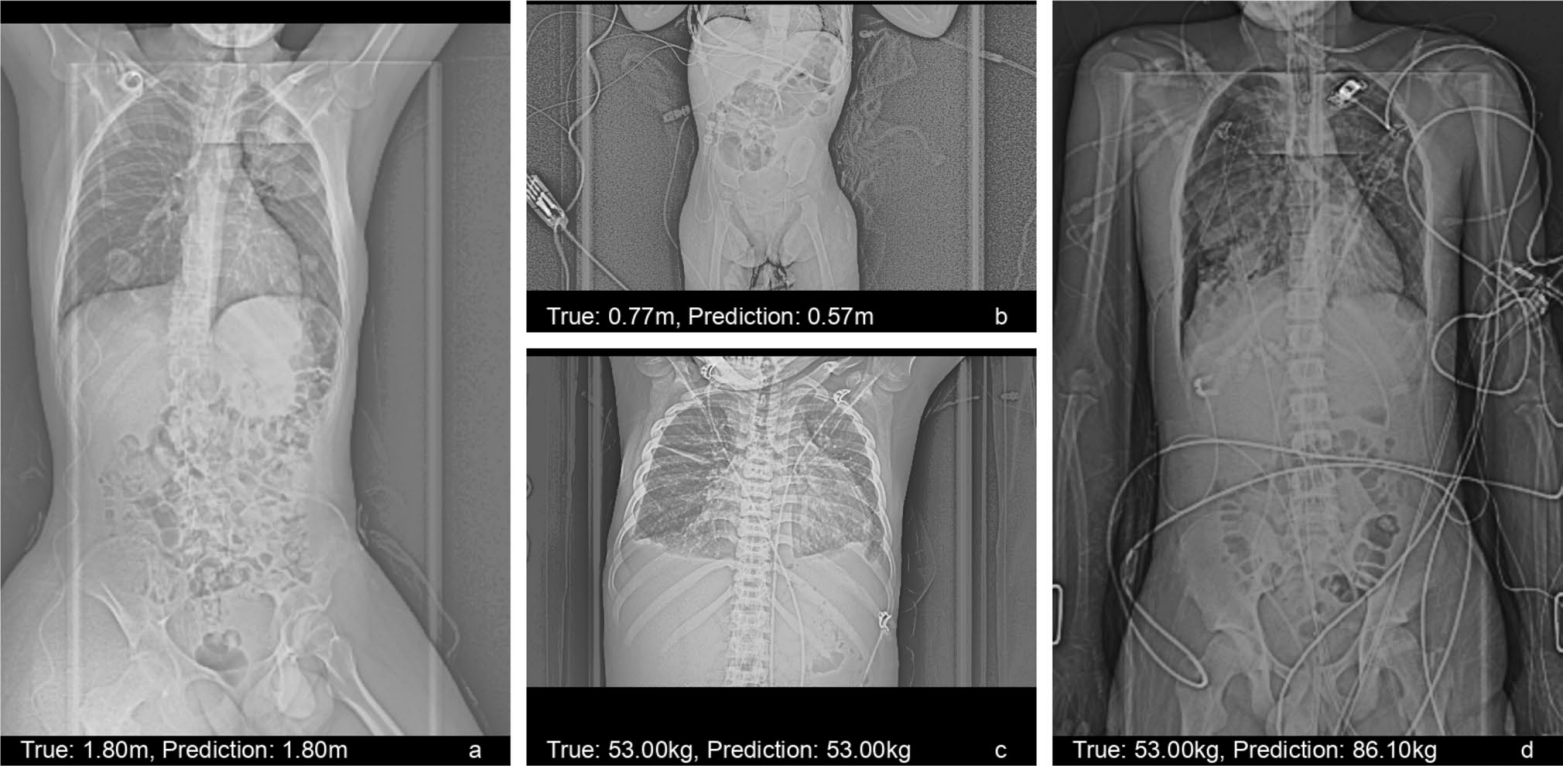
Example CT localizers with best and worst predictions. (**a**) Prediction with lowest height error. (**b**) Prediction with highest height error. (**c**) Prediction with lowest weight error. (**d**) Prediction with highest weight error.

For predicting the body height, the models showed an MAE of 5.58 ± 4.26 cm, and for the body weight of 4.25 ± 4.28 kg, with correlation coefficients of R = 0.982 and R = 0.978. The relative mean error was 4.12 ± 4.05% for height and 11.28 ± 12.05% for weight. The performance of the final models was rather similar to the performance observed on the validation set, although a drop in performance could be seen, hinting at some overfitting in the training process. The prediction errors were generally relatively small (Fig. 4a,d), and only a few very large could be seen (Fig. 4b,e). There was no clear association between height and weight error, however, for smaller and heavier patients the prediction seemed to be worse (Fig. 4c,f). The best and worst predictions for body height and weight are visualized in Fig. 5.

## Discussion

Assessment of somatometric parameters based on radiological scans has been considered for a long time^5–8^. Our results showed that the prediction of body weight and height from CT localizers can automatically be performed in pediatric patients with high accuracy using deep learning methods.

The best-performing models achieved a mean absolute error around 6 cm for predicting the body height and 4 kg for predicting the body weight in the independent test set. While in machine learning it is often standard to measure absolute errors, in clinical routine the relative errors are more important. Here, our model achieved an error of 4% for body height, but a larger error of 11% for body weight. This result seems to contradict the absolute errors at first sight, since prediction of body weight performed better in terms of MAE, however, the discrepancy stems from the different distributions (Fig. 2). Furthermore, it is reasonable that height can be predicted better than weight since the localizers were normalized such that a pixel corresponds to 1 mm. A similar normalization is not possible for weight. From a clinical perspective, the errors can be regarded to be within acceptable limits^14^, even though a few outliers were observed.

In addition, a slight overfitting was visible in the test cohort when compared to the predictions on the validation cohort. We suspect that a larger sample size could lead to better model performance. This can be seen rather clearly for patients with greater body weight, where only few samples were available (Fig. 2) and the predictions showed greater variation there (Fig. 4f).

We used a hyperparameter optimization framework to develop our models, which showed that the EfficientNetV2 performs better than the ResNet-based networks, which is in line with other studies^15, 16^. In addition, since CT localizers with corresponding height and weight measurements are relatively infrequent in our hospital, we tested whether CT localizers from younger adults can improve the overall accuracy. Indeed, this was the case, although the difference was relatively low, with an improvement of 0.39 cm for height and 0.22 kg for weight.

Our study focused on pediatric patients, but a similar automation for adult cohorts has been performed recently by Ichikawa et al. to estimate the weight of a patient^13^. In contrast to our study, they distinguished between chest and abdominal CT localizers. Their results show an MAE of 2.75 kg for thoracic and of 4.77 kg for abdomen localizers. However, since the cohort consisted of adults older than 24 years, the question if such an approach can also be used for pediatric patients, was open. Our study showed that this is indeed the case, and the overall accuracy was comparable since our model was using thoracic as well as abdomen localizers.

We employed CT localizers, whose usefulness to optimize the CT protocol has recently been explored; for example, they are utilized to predict the organ-level radiation dose^17^, water-equivalent diameter^18^, or the optimal scan range^19^. However, weight and height assessments can be performed in different modalities as well. In a large-scale study, Langner et al. used a deep neural network to predict height and weight in adult patients using neck-to-knee MRIs^8^. Their results showed an MAE of 1.70 cm for height and of 0.78 kg for weight. This excellent accuracy might be explained by the large and more homogenous patient cohort, which comprised over 32,000 healthy volunteers, allowing the network to generalize better.

Using CT scans, Geraghty et al.^6^ estimated weight, height, body mass index, and body surface area from a single axial slice of abdominal CT scans from adult patients. Their manual method consists of outlining the first lumbar vertebra on the most central slice through L1. Using a linear model, they reported a correlation of R = 0.93 for weight and R = 0.65 for height. Similarly, Zopfs et al.^5^ determined the patient’s body weight and body composition using a single axial CT slice at the height of the third lumbar vertebra. For this, they manually measured the areas of paraspinal muscles and employed a linear model for prediction, which used body height as an independent variable. They reported an adjusted R^2^ of 0.886 for the model. Since both studies were only performed for adult patients, a direct comparison to our results is not possible. While they seem comparable, the advantage of our method is the automatic assessment which does not require manual labeling.

A strength of our method is that it could be easily be integrated into the clinical routine since the estimation is based on CT localizers required for any CT scan. In addition, we used all available data with no exclusion criteria based on any pathology. Therefore, we expect the algorithm to perform well at our site, even when used in a prospective context like in emergencies. It could potentially be used to indicate radiation dose excess of weight-based DRLs before the CT scan and calculate accurate amounts of contrast material based on weight.

Limitations apply to our study: In routine clinical practice, body measurements are not always accurate since, at times, these are not taken but either guessed by the technician or inquired from the patient or accompanying guardians. In routine clinical practice, more accurate measurements sometimes cannot be taken, e.g., in case of patients confined to bed. Excluding such patients would introduce a bias since the network would be trained only on more healthy patients. Since neural networks can learn through noise^20, 21^, we believe that the network is accurate nonetheless. In addition, for many patients, multiple readings were available, and we ensured that the measurements were not contradicting.

Another limitation concerns the positioning of the patient in the CT gantry. If the patient is not aligned accurately to the isocenter of the table, the patient might appear larger or smaller on the CT localizer^22^. This effect is larger for pediatric patients since they are smaller, have different body proportions than adults and their positioning is more challenging^23–26^. Since our model cannot not directly account for these errors, a bias might occur if the position of a patient is different than those in the training set.

We also did not distinguish between thoracic and abdominal localizers since, in clinical routine, there is an inevitable overlap between both. While a more refined dataset could increase the accuracy^13^, the sample sizes would decrease, which in turn could hurt the networks’ performance. Since obtaining a larger data set was not feasible in our case, we included readings within 3 months of the acquisition of the localizer to increase the sample size. Still, this approach increases the uncertainty if a disease affects the patient’s weight. Our model was developed on CT localizers acquired at a single site, on different scanner models from a single vendor. Although CT localizers are relatively homogenous from an imaging point of view, the model should be tested in a future study on external data acquired on scanners from other vendors.

In conclusion, we presented an automated assessment of body height and weight based on CT localizers from pediatric patients which showed an overall high accuracy.

## Methods

Ethical approval for this retrospective study was granted by the local ethics committee (Ethics Commission of the Medical Faculty of the University of Duisburg-Essen; registry number 21-10069-BO). Written and informed consent was waived by the Ethics Commission of the Medical Faculty of the University of Duisburg-Essen because of the retrospective nature of this study. All experiments were conducted in accordance with the relevant guidelines and regulations.

### Patient cohorts

CT examinations of the thorax or abdomen that included a CT localizer were collected anonymously via a query in our hospital’s radiological information system (RIS). Three independent data sets were created: First, a training set to train neural networks, a validation set to optimize the hyperparameters of the trained models, and a test set used only once after the entire training was completed to estimate the accuracy of the final trained model.

The training set included all examinations between January 2013 and December 2019 of pediatric patients (< 21 years) for each CT procedure. Localizers with pixel spacing less than 1 mm or greater than 2 mm (indicating either phantom or acquisition error) were removed. Localizers with a patient position other than head-first-supine or missing sex information were removed, indicating a phantom or a different CT examination.

Similarly, the validation set comprised examinations between January and December 2020, while the test set consisted of examinations between January 2021 and March 2022. Care was taken to ensure that each cohort was separate, i.e., all patients appeared only in the training, test, or validation set. If multiple localizers were available for a patient in the validation or test sets, only the chronologically first was retained after applying the exclusion criteria because multiple localizers from a single could lead to statistical bias.

Furthermore, a training set of younger adults (aged from 21 to 40 years) between January 2013 and December 2013 was acquired. The rationale for collecting this set is that it might benefit the neural network during training since it increases the overall sample size. This set was similarly preprocessed as the training cohort.

### Outcomes

Body height and weight were collected from the DICOM data of the CT localizer. In case of missing outcomes, the hospital-internal Fast Healthcare Interoperability Resources (FHIR) server was queried using the Health Level Seven International (HL7) standard. This server is a hospital-wide resource storing patient-related information generated during the clinical routine. However, since measurements from this server might have been performed at a different time than the acquisition of the CT localizer, only measurements within three months of the acquisition date were used. If multiple measurements fulfilled this restriction, the closest one was used.

In a few cases, unreasonable heights and weights (difference more than 10 kg and more than 10 cm) were observed. These cases were fixed by counter-checking the DICOM and FHIR resources where possible. Measurements were removed if and only if an apparent contradiction was observed. In the cases where the accurate measurement could be inferred (e.g., if a number twist occurred), the measurements were corrected.

### CT localizer acquisition parameters

CT scans were performed on scanners from a single vendor (Siemens Healthineers). Corresponding CT localizers were acquired in inspiration, as far as patients were able to do so, in the anterior–posterior direction with a tube voltage varying between 80 and 140 kV and tube currents between 20 and 110 mA (Table 2).

**Table 2:** CT scanners used for the acquisition of the CT localizers.

Scanner			Height			Weight		
	Tube voltage	Tube current	Train (N = 1009)	Validation (N = 116)	Test (N = 203)	Train (N = 1111)	Validation (N = 127)	Test (N = 225)
SOMATOM force	120 kV	20 mA	374	71	45	407	76	50
SOMATOM definition flash	120 kV	35 mA	413	12	47	446	13	53
SOMATOM definition AS +	120 kV	36 mA	147	15	18	163	16	21
SOMATOM definition edge	120 kV	35 mA	0	3	70	0	6	77
SOMATOMVolume zoom	120 kV	100 mA	54	0	0	67	0	0
SOMATOM definition AS	120 kV	35 mA	6	13	23	8	14	24
Other	80–140 kV	20–60 mA	15	2	0	20	2	0

Scanners with less than 50 examinations were subsumed under the “Other” group.

### Preprocessing

Intensities of all CT localizers were first linearly rescaled; then, contrast-limited adaptive histogram equalization (CLAHE)^27^ was applied to increase the contrast. Pixel spacings were homogenized by rescaling them to a spacing of 1 mm and padded (or cropped) to the size of 768 × 512 pixels. For more details, refer to the Supplementary information.

### Neural network training

We employed three different networks which used the whole image to predict the body height and weight directly. All networks were pretrained on the ImageNet dataset^28^. Augmentations were applied during training to virtually increase the sample size (more details can be found in the Supplementary information).

Successful training of a neural network depends critically on the choice of hyperparameters, for example, the network architecture and the learning rate. Since choosing these is generally tricky, we employed a tuning framework, Optuna^29^. This framework optimized several network hyperparameters: the backbone architecture, the network head, the number of trainable layers and the learning rate (see Supplementary information). The training was performed by minimizing the L2 loss using the AdamW optimizer^30^. To further increase the performance of the network, small random image transformations, also called augmentations, were applied to all CT localizers. The validation set was used for early stopping since this is known to prevent overfitting.

The training was conducted using Python 3.10 and Pytorch 2.0. The code for training the network and evaluation will be made available on GitHub (https://www.github.com/aydindemircioglu/scout.view.height.weight).

### Adult training cohort

In addition to the training set, we prepared a second set containing CT localizers of younger adults (between 21 and 40 years). The rationale for using this set is that including them in the training set could improve the training since more variation is captured by the additional data, possibly improving the overall accuracy. For example, younger adults might show more signs of obesity, which is relatively rare among children. Therefore, in addition to the training with pediatric patients only, a second training was performed, including the adult training set, which roughly doubled the training sample size.

### Validation

The quality of the network prediction was measured by the absolute mean error (MAE) on the validation set. In addition, mean absolute percentage error (MAPE) and the Pearson correlation coefficient was computed.

The better of the two training strategies, one using the adult training cohort and the other without, were then selected based on the MAE in the validation cohort. Two models were then retrained on all data comprising the training data and the validation data, with the hyperparameters of the best-performing models. These two models were then regarded as the final models. The quality of these models was then evaluated once with the same metrics on the independent test cohort. In addition, the Pearson correlation coefficient was computed to judge the linear relationship between predictions and the actual outcomes.

### Supplementary Information

Supplementary Information.

## Data Availability

The datasets generated during and/or analysed during the current study are available from the corresponding author on reasonable request. The code for training the network and evaluation will be made available on GitHub (https://www.github.com/aydindemircioglu/scout.view.height.weight).

## Abbreviations

CLAHE: Contrast-limited adaptive histogram equalization
DICOM: Digital imaging and communications in medicine
DRL: Diagnostic reference level
FHIR: Fast healthcare interoperability
HL7: Health level seven international
MAE: Mean absolute error
MAPE: Mean absolute percentage error
RIS: Radiological information system
