# Supplementary material for: Determining body height and weight from thoracic and abdominal CT localizers in pediatric and young adult patients using deep learning
Source: Sci Rep. 2023 Nov 3;13:19010. doi: 10.1038/s41598-023-46080-5 (PMC10624655; doi:10.1038/s41598-023-46080-5)
Supplement: Supplementary file 1 — Supplementary Information. [file 41598_2023_46080_MOESM1_ESM.docx]

**Supplemental Information**

**Determining body height and weight from thoracic and abdominal
CT localizers in pediatric patients using deep learning**

***Demographics of the training cohorts***

|  | **All (N = 1184)** | **Training (N = 537)** | **Adult training (N = 647)** |
| --- | --- | --- | --- |
| **Gender (Female/Male)** | 42.2% (510/1184) | 42.3% (227/537) | 43.7% (283/647) |
| **Height [m]** | 1.62 +/- 0.29 | 1.47 +/- 0.36 | 1.74 +/- 0.1 |
| **Weight [kg]** | 62.8 +/- 26.7 | 48.2 +/- 27.0 | 74.9 +/- 19.3 |
| **Age [years]** | 22.3 +/- 10.3 | 13.3 +/- 6.8 | 29.8 +/- 5.4 |

**Table S1** Demographics of the training cohorts.

#### ***Preprocessing***

All CT localizers were converted from the DICOM format to PNG. As the CT localizer is a 16-bit image that contains absolute HU values, which might be necessary to estimate the body height and weight, only global rescaling was applied. First, it was ensured that no values below -256 or above 1024 appeared by clipping. Then the image was linearly rescaled to the range (0, 255). Next, to homogenize the pixel spacing, all localizers were rescaled to a spacing of 1 mm. The images were then cropped symmetrically to the size of 768 x 512 pixels. After this, the contrast of the localizers was increased using contrast-limited adaptive histogram equalization (CLAHE)^1^ with two different parameter sets (clipLimit of 32 and 64, tileGridSize of (2,2) and (1,1) resp.) and added to the image to obtain an RGB image.

***Neural Network architecture***

All networks considered consisted of a backbone and a fully connected head. The head replaced the final classification layers of the backbone and comprised three fully connected layers. Between the backbone and the head, a dropout layer (with fixed dropout of 0.1) was applied. The L2 loss was used since this loss tends to avoid larger prediction errors which would be detrimental to clinical routine. It was optimized using the AdamW optimizer (with default values betas = (0.9, 0.999) and a weight decay of 0.01). The batch size was chosen as large as feasible, which was 16 or 32. The maximum number of epochs was fixed at 100. Early stopping was used to prevent overfitting and training was stopped when the decrease in the validation loss was less than 0.05 over 20 epochs.

To further increase the performance of the network, random image transformations, also called augmentations, were applied to all CT localizers. However, care was taken that no transformation would alter the image with respect to the outcome. For example, random resizing or cropping of the image would be harmful to predicting body height and weight since the patient would appear larger or smaller than in reality. On the other hand, small rotations would not impede the prediction. Therefore, only small random rotations (between -5 and 5 degrees), slight random brightness and contrast changes (± 10%), and small random erasing with a black box (with a size < 15%) were applied to the images.

***Hyperparameter Optimization***

The neural network architecture and its training depend on several hyperparameters one must choose appropriately. Since choosing these is generally tricky, we employed a tuning framework, Optuna, a hyperparameter optimization library based on Tree Parzen Estimators, was chosen for efficient tuning^2^. In detail, the following parameters were optimized by Optuna:

- The choice of network backbone (ResNet-18, ResNet-34, EfficientNetV2-S)
- The size of the three fully connected layers of the head (each between 8, 16, …, 1024)
- The initialization of the weights of the layers and the freezing of them (determined both by a single number between -1 and 4; -1 = use random weights, 0 = use pretrained weights, between 1 and 4 = use pretrained weights and freeze parts of the network)
- The learning rate (log-uniform in the range 0.01 and 1e-5)
- The learning rate schedule (gamma in 0.1, 0.2, …, 1.0 and step size in 15, 16, …, 30).

Scheduling was performed by multiplying the learning rate with the chosen gamma after a given number of epochs (called step-size scheduling). Finally, the freeze parameter was optimized, which determined how many parameters of the backbone were trainable or fixed during training. For this, the layers of each network architecture were roughly divided into four parts. In more detail (using the naming conventions of the respective studies by He et al.^3^ and Huang et al.^4^):

- For the EfficientNet V2-S: Freeze = 1 froze stage 0 and stage 1. Freeze = 2 froze in addition stage 2 and 3. Freeze = 3 froze in addition stage 4 and 5. Freeze = 4 froze all layers.
- For the ResNets: Freeze = 1 froze convolutional+pooling layers and conv2_x. Freeze = 2 froze in addition conv3_x. Freeze = 3 froze in addition conv4_x. Freeze = 4 froze all layers.

Weights of fully connected layers were initialized using the method by He et al.^5^ and were always trainable. A full overview of the amount of trainable and non-trainable parameters can be found in Table S2.

| **Freeze level** | **Parameter** | **ResNet-18** | **ResNet-34** | **EfficientNet V2-S** |
| --- | --- | --- | --- | --- |
| 0 | Trainable | 11,176,512 | 21,284,672 | 20,177,488 |
|  | Non-trainable | 0 | 0 | 0 |
| 1 | Trainable | 11,019,008 | 21,053,184 | 20,166,328 |
|  | Non-trainable | 157,504 | 231,488 | 11,160 |
| 2 | Trainable | 10,493,440 | 19,936,768 | 19,273,592 |
|  | Non-trainable | 683,072 | 1,347,904 | 903,896 |
| 3 | Trainable | 8,393,728 | 13,114,368 | 14,892,072 |
|  | Non-trainable | 2,782,784 | 8,170,304 | 5,285,416 |
| 4 | Trainable | 0 | 0 | 0 |
|  | Non-trainable | 11,176,512 | 21,284,672 | 20,177,488 |

**Table S2** Parameter count for the pretrained backbones of the network architectures. The freeze level refers to the layers that were frozen during training. A freeze level of 0 will not freeze any layer, while a freeze level of 4 will freeze all weights of the backbone; only the network head with fully connected layers will be trainable in this case.

The total rounds of parameter optimization to be searched by Optuna were fixed to 100. In each optimization round, Optuna selected the hyperparameters of the network, which were then trained on the training data set and evaluated on the validation set by computing the MAE. After optimization, the hyperparameters of the best-performing model in terms of MAE was chosen as the final model.

After the best network structure was determined, the network was trained using both training and validation data sets. This is because, in general, neural networks benefit from more data. The training was conducted as many epochs as during the training of the best model. The retrained model was considered to be the final model. Its performance was then evaluated on the independent test data set. This final evaluation took only place once to avoid introducing any bias by repeatedly optimizing for the test set, which would lead to severe overfitting.

***Software***

The neural network was developed using the Python 3.10, Pytorch 2.0^6^. For reproducibility, the code for training the neural network and evaluation will be made available on GitHub (<https://github.com/aydindemircioglu/scout.view.height.weight>).

***References***

1. Pizer, S. M. *et al.* Adaptive histogram equalization and its variations. *Comput. Vis. Graph. Image Process.* **39**, 355–368 (1987).

2. Akiba, T., Sano, S., Yanase, T., Ohta, T. & Koyama, M. Optuna: A Next-generation Hyperparameter Optimization Framework. *ArXiv190710902 Cs Stat* (2019).

3. He, K., Zhang, X., Ren, S. & Sun, J. Deep Residual Learning for Image Recognition. in *2016 IEEE Conference on Computer Vision and Pattern Recognition (CVPR)* 770–778 (2016). doi:10.1109/CVPR.2016.90.

4. Huang, G., Liu, Z., van der Maaten, L. & Weinberger, K. Q. Densely Connected Convolutional Networks. *ArXiv160806993 Cs* (2016).

5. He, K., Zhang, X., Ren, S. & Sun, J. Delving Deep into Rectifiers: Surpassing Human-Level Performance on ImageNet Classification. in 1026–1034 (2015).

6. Paszke, A. *et al.* PyTorch: An Imperative Style, High-Performance Deep Learning Library. *ArXiv191201703 Cs Stat* (2019).
